# The role of circadian phase in sleep and performance during Antarctic winter expeditions

**DOI:** 10.1111/jpi.12817

**Published:** 2022-07-25

**Authors:** Tracey L. Sletten, Jason P. Sullivan, Josephine Arendt, Lawrence A. Palinkas, Laura K. Barger, Lloyd Fletcher, Malcolm Arnold, Jan Wallace, Clive Strauss, Richard J. S. Baker, Kate Kloza, David J. Kennaway, Shantha M. W. Rajaratnam, Jeff Ayton, Steven W. Lockley

**Affiliations:** ^1^ Turner Institute for Brain and Mental Health and School of Psychological Sciences Monash University Victoria Australia; ^2^ Division of Sleep and Circadian Disorders, Departments of Medicine and Neurology Brigham and Women's Hospital Boston Massachusetts USA; ^3^ Faculty of Health and Medical Sciences University of Surrey Guildford Surrey UK; ^4^ Suzanne Dworak‐Peck School of Social Work University of Southern California Los Angeles California USA; ^5^ Division of Sleep Medicine, Harvard Medical School Boston Massachusetts USA; ^6^ Polar Medicine Unit, Australian Antarctic Division Kingston Tasmania Australia; ^7^ Robinson Research Institute, School of Medicine, Discipline of Obstetrics and Gynaecology University of Adelaide Adelaide South Australia Australia

**Keywords:** Antarctica, circadian, melatonin, performance, sleep, space analog

## Abstract

The Antarctic environment presents an extreme variation in the natural light‐dark cycle which can cause variability in the alignment of the circadian pacemaker with the timing of sleep, causing sleep disruption, and impaired mood and performance. This study assessed the incidence of circadian misalignment and the consequences for sleep, cognition, and psychological health in 51 over‐wintering Antarctic expeditioners (45.6 ± 11.9 years) who completed daily sleep diaries, and monthly performance tests and psychological health questionnaires for 6 months. Circadian phase was assessed via monthly 48‐h urine collections to assess the 6‐sulphatoxymelatonin (aMT6s) rhythm. Although the average individual sleep duration was 7.2 ± 0.8 h, there was substantial sleep deficiency with 41.4% of sleep episodes <7 h and 19.1% <6 h. Circadian phase was highly variable and 34/50 expeditioners had sleep episodes that occurred at an abnormal circadian phase (acrophase outside of the sleep episode), accounting for 18.8% (295/1565) of sleep episodes. Expeditioners slept significantly less when misaligned (6.1 ± 1.3 h), compared with when aligned (7.3 ± 1.0 h; *p* < .0001). Performance and mood were worse when awake closer to the aMT6s peak and with increased time awake (all *p* < .0005). This research highlights the high incidence of circadian misalignment in Antarctic over‐wintering expeditioners. Similar incidence has been observed in long‐duration space flight, reinforcing the fidelity of Antarctica as a space analog. Circadian misalignment has considerable safety implications, and potentially longer term health risks for other circadian‐controlled physiological systems. This increased risk highlights the need for preventative interventions, such as proactively planned lighting solutions, to ensure circadian alignment during long‐duration Antarctic and space missions.

## 1 INTRODUCTION

Ocular light exposure is the primary environmental time cue for synchronizing the circadian pacemaker.^1^ Inappropriate or absent exposure to a 24‐h light‐dark cycle causes misalignment of sleep and wake timing relative to the circadian pacemaker when sleep timing is unchanged, resulting in disruptions to sleep quality and duration, impaired performance, and disrupted metabolic and endocrine systems.^2^, ^3^ The Antarctic environment presents an extreme seasonal variation in the natural light‐dark cycle, with near‐constant sunlight during summer, and low levels of external light during the winter.^4^, ^5^, ^6^ During the Antarctic winter, the diminished intensity of natural light exposure and shortened photoperiod, combined with insufficient electric light provision,^5^, ^7^, ^8^ leads to seasonal shifts, often delay shifts, in circadian phase.^6^, ^8^, ^9^, ^10^, ^11^, ^12^ Rarely is the available light insufficient to maintain circadian synchronization to the 24‐h day, however.^4^, ^13^

The strong phase relationship between the circadian system and the timing of sleep and wake^14^ dictates that optimal sleep occurs during the biological night (the duration of melatonin secretion) and that sleeping outside of this window is associated with reduced duration and quality of sleep.^15^ Although a delay in circadian phase is often experienced during the Antarctic winter, a concomitant delay in the timing of expeditioner sleep often cannot occur due to imposed station schedules, and consequently sleep is placed at a suboptimal circadian phase, causing expeditioners to wake too early in their circadian cycle.^11^ Sleep disruption is one of the most common difficulties reported by expeditioners wintering at Antarctic stations,^5^, ^7^, ^16^, ^17^ including reductions in total sleep time, sleep efficiency, and slow wave sleep.^7^, ^17^, ^18^

Mood is also under strong circadian and sleep‐wake‐dependent control.^19^ Disruptions to circadian rhythms and sleep are therefore associated with disturbances in mood in temperate environments^20^ and impairments in affect may be exacerbated by the extremely isolated conditions of Antarctica. Impaired mood and negative affect during the winter season in Antarctica have been reported, including increases in symptoms of depressed mood, anger, and irritability^12^, ^16^, ^21^, ^22^ and interpersonal tension and conflict toward team members.^23^ A peak in negative symptoms soon after midwinter, often in the third quarter of the expedition has been identified^24^ although not in all expeditions.^25^, ^26^, ^27^

Only a few studies have directly examined the role of circadian misalignment on sleep, cognitive performance, and mood during the Antarctic winter.^12^, ^28^, ^29^ Research in the Antarctic environment has been restricted by the small number of expeditioners that overwinter at a single station, which does not account for large individual variability.^30^, ^31^ Due to known interindividual differences in the phase angle between circadian phase and sleep‐wake timing under normal conditions,^32^, ^33^ the response to sleep loss,^34^, ^35^, ^36^ and direction and rate of circadian adaptation,^37^, ^38^ examination of larger populations is necessary. This study examined the impact of wintering in Antarctica on circadian rhythms and sleep duration across multiple stations and assessed the role of changes in the relative timing of sleep and the circadian pacemaker on psychophysiological responses of cognitive functioning and mood disturbances.

## 2 MATERIALS AND METHODS

### 2.1 Participants

Participants were 64 expeditioners based at three Australian Antarctic Research Stations during two Austral winter seasons; Davis (68°34'S,77°58'E), Mawson (67°36'S,62°52'E), and Casey (66°16'S,110°31'E). During the Austral winter, the sun remains below the horizon for 38, 16, and 0 days at Davis, Mawson, and Casey stations, respectively. All expeditioners over‐wintering at these stations were invited to participate (*n* = 95) and acceptance to the expedition was considered a sufficient inclusion criterion. Further conditions such as ocular health or color vision were not specifically assessed for participation. Color vision was not specifically assessed in all individuals before expedition. Over half of the expeditioners (54.9%) were taking dietary supplements, particularly vitamin D. Two participants (3.9%) were on medications for high cholesterol and gastrointestinal reflux.

Of 64 initial volunteers, 9 voluntarily withdrew before data collection, 2 did not over‐winter, and 2 withdrew due to scheduling conflicts with the study procedures. Data from 51 participants were available for analyses (*n* = 50 for subjective sleep data), representing 54% (51/95) of personnel over‐wintering at the three stations during the study duration. Expeditioners included in the analyses (41 M, 10 F) were 45.5 ± 11.9 years (mean ± SD, range 27–69 years) and had a mean body mass index of 26.3 ± 5.6 kg/m^2^ (Table 1). Participants provided written informed consent and study procedures were approved by the Partners Human Research Committee, Monash University Human Research Ethics Committee, Committee for Protection of Human Subjects at NASA Johnson Space Center, and the Australian Antarctic Program Human Research Ethics Committee. The procedures were performed according to the principles outlined by the Declaration of Helsinki.

**Table 1:** Demographic summary for participating expeditioners

	***n***	**Mean** ± **SD**
Total expeditioners	51	
n per station		
Year 1: Davis	15	
Year 1: Mawson	8	
Year 1: Casey	8	
Year 2: Davis	8	
Year 2: Mawson	12	
Sex (M, F)	41, 10	
Age (n; y)	51	45.50 ± 11.86
Body Mass Index (n; kg/m^2^)	41	26.29 ± 5.60
Mean total sleep time, subjective (n; h)	47	7.20 ± 0.78
Mean total sleep time, actigraphic (n; h)	23	6.47 ± 1.53

### 2.2 Procedures

Data collection occurred during the winter Antarctic expedition season. The exact data collection start and end dates differed for each station, based on station arrival and departure schedules but typically occurred from March to September. Data included assessments of circadian phase, sleep, work, psychological health, and cognitive performance.

#### 2.2.1 Sleep

Expeditioners completed a daily sleep diary to record bed and rise times, sleep onset latency, and the number and duration of awakenings. A subset of participants (*n* = 23) also wore a wrist activity monitor (Actiwatch Spectrum, Philips Respironics). Participants wearing an activity monitor were selected by station doctors without specific inclusion criteria applied. Activity monitors were worn on the nondominant wrist throughout the study. Sleep was examined using Actiware 5.0 software (Philips Respironics). Sleep diary data from one participant were excluded from analyses due to poor record keeping. A total of 6775 days of sleep log data were collected. Of these sleep episodes, actigraphic assessment of sleep was available for 2566 days.

#### 2.2.2 Circadian phase

Circadian phase was assessed in all participants from the rhythm of the urinary melatonin metabolite, 6‐sulphatoxymelatonin (aMT6s). Once a month, participants collected urine sequentially every 3–4 h (6–8 h when asleep) over 48 h. Participants recorded the time and volume of each collection and preserved a 5 ml sample at –20°C until the end of the season. Urine samples underwent radioimmunoassay to determine the concentration of aMT6s.^39^ Assays were completed at the University of Adelaide with reagents supplied by Stockgrand, Ltd, University of Surrey. All samples from an individual expeditioner were included in the same assay. The interassay coefficients of variation at 3.3, 14.0, and 29.0 ng/ml were 15.3%, 14.6%, and 15.5%, respectively. The median intraassay coefficient of variation was 6.8%. The limit of detection of the assay was 0.5 ng/ml.

Urinary aMT6s excretion rate (ng/h for each collection episode, timed according to the midpoint of the collection) was analyzed by cosinor analysis to calculate the predicted peak time (acrophase) of the rhythm, a procedure used extensively in field settings, including Antarctica.^9^, ^29^, ^40^ Regression analyses of significant acrophases were conducted^41^ and results that showed a significant fit to a cosine curve (*α* set at.10) were used in further analyses (*n* = 263, 88% were *p* < .05). Adjustments were made to analysis methods when there were errors in urine collection. In 11/323 48‐h blocks, the first data point was removed before cosinor analyses due to participants including the first morning void in the first sample collected. On four 48‐h blocks, the last data point was removed. Of a possible 357 collections (51 participants with a maximum of 7 × 48‐h collections), 318 48‐h collections were obtained (3090 samples), and 263 acrophase times calculated.

#### 2.2.3 Cognitive assessments

For 48‐h per month, coinciding with the timing of urine collection, participants completed computer‐based tests of cognitive performance and mood three to four times across the day using the Automatic Neuropsychological Assessment Metric‐Isolated and Confined Environments (ANAM‐ICE). ANAM‐ICE was developed specifically for the Antarctic environment, with adaptations from tests developed by the Department of Defense.^42^ Tests presented in the ANAM‐ICE battery were the Code Substitution Delayed Memory test^43^ and the Memory Search task.^43^ Over 93% of monthly test bouts began within 1 day of the urine collection start date (0.38 ± 1.47 days from the first day of urine collection). ANAM‐ICE also included subjective measures of affect. Following the objective performance tests, participants were presented with a selection of adjectives related to affect and selected a number that best described their current state on a scale of 0 = not at all and 6 = very much. Further details on cognitive assessments are presented in Supporting Information.

#### 2.2.4 Psychological health assessments

Questionnaires to assess health, mood, and team interactions were completed at the start and end of the expedition, and each month during the over‐winter. Questionnaires were completed online using a secure web‐based application with an individual link emailed directly to each participant every month. Survey data were collected and managed using REDCap (Research Electronic Data Capture) electronic data capture tools.^44^ Of the 311 blocks of online surveys sent to participants, 231 blocks were completed (74%). At the beginning and end of the season, the Morningness/Eveningness Questionnaire (MEQ)^45^ was used to evaluate diurnal preference.

Monthly assessments included the short (30‐item) form of the Profile of Mood States (POMS‐SF),^46^ the Positive Affect Negative Affect Schedule (PANAS),^47^ the Depression Anxiety Stress Scale (DASS),^48^ the Patient Health Questionnaire (PHQ), completed as an assessment of depression,^49^ the Perception of Current Conflict (PCC) scale^50^ and the Team Member Exchange Questionnaire.^51^ Further details on psychological health assessments are presented in Supporting Information.

### 2.3 Data analyses

#### 2.3.1 Sleep and wake

The distributions of subjective total sleep time and duration of time awake between sleep episodes were examined for 47 participants with three participants removed for contributing fewer than 10 episodes of sleep or wake. Subjective total sleep time was calculated as time in bed minus sleep onset latency and wake duration after sleep onset, until rise time. Where sleep onset latency or wake duration were not recorded (256/6775 logs), the sleep episode was excluded from total sleep time calculations (total sleep time *n* = 6519 sleep episodes). Sleep and wake episodes were examined separately as the average duration for each participant and the duration for all episodes across the season.

#### 2.3.2 Circadian phase and circadian misalignment

Daily circadian phase for 5 days on either side of each monthly acrophase calculation was estimated by extrapolation between measured aMT6s acrophases.^41^ The phase angle of entrainment between acrophase and subjective sleep offset was calculated by subtracting aMT6s acrophase from sleep offset time. Interpolated acrophase time was aligned with the timing of each sleep episode to identify whether the acrophase occurred within or outside of the sleep episode, categorizing each sleep episode as aligned (in‐phase) or misaligned (out‐of‐phase), respectively. The approach of using an established marker of circadian phase occurring within the sleep episode to define circadian alignment has been applied previously.^52^ For each participant contributing data to both aligned and misaligned sleep episodes, mean subjective total sleep time was calculated separately for aligned and misaligned sleep. Paired *t* tests were applied to compare subjective total sleep time for aligned and misaligned sleep. *χ* ^2^ tests were applied to examine whether the misalignment of sleep was associated with obtaining <7 h and <6 h of sleep. In a subset of participants (*n* = 23), actigraphic total sleep time was compared for aligned and misaligned sleep episodes to confirm the results for subjective sleep (see Supporting Information).

#### 2.3.3 Cognition, mood, and psychological health

ANAM‐ICE cognitive performance and mood outcomes (*z*‐scored) were analyzed relative to the duration of wake and circadian phase. Time of testing was represented by time since waking from a sleep episode (hours) and assigned to six 3‐h bins. The time of test relative to aMT6s acrophase (hours) was assigned to seven 3‐h bins (the eighth bin contained no data). Mixed model analyses of variance were conducted (SAS Institute, version 9.4).

The 25th and 75th percentiles of phase angle on the night before ANAM‐ICE tests were used to assess the impact of circadian misalignment on daytime performance and mood. Temporal changes in performance and mood outcomes relative to time since waking were compared for tests following 25% most advanced phase (phase angle at sleep offset 3.1 –6.8 h, *n* = 102 tests) and 25% most delayed phase (phase angle at sleep offset 0.4 h to −7.9 h, n = 84 tests) for expeditioners contributing data to both groups (*n* = 13).

Psychological surveys were grouped by month, based on the completion date, to examine changes across the season. Participants completing fewer than three of six possible monthly surveys were excluded from analyses. Analyses included surveys from 43 expeditioners. For each month the proportion of scores above the predetermined clinically relevant threshold for each survey was calculated. Linear mixed models were applied to examine changes across the season.

To assess the potential influence of time into expedition on psychological health, independently of timing within the winter season (i.e., differences in photoperiod),^17^, ^28^ subset analyses compared expeditioners who arrived at the station at the start of the winter participation period (*n* = 14), compared with expeditioners who were also on station for the prior Austral summer (*n* = 17). Linear mixed models compared psychological surveys based on time on station (winter only vs. summer and winter) and point of assessment (early in winter vs. late in winter).

## 3 RESULTS

Of the 46 participants who completed a baseline survey at the start of their participation, 63.0% identified it was their first winter deployment in Antarctica; seven of these had completed at least one summer deployment. The remaining 37.0% had completed at least one over‐winter and at least one summer deployment. The majority of participants (71.7%) arrived in Antarctica at least 2 months before completing their baseline survey and almost half (47.8%) had arrived at least 4 months prior. The majority of participants had regular duty responsibilities starting between 07:30 h and 08:00 h with a work duration of 9–12 h. Very few participants had alternative duty hours in line with their specific role and operational requirements: some individuals started duty before 06:00 h and could work extended duty and few frequently worked hours during the night.

### 3.1 Sleep and wake

**Figure 1:**
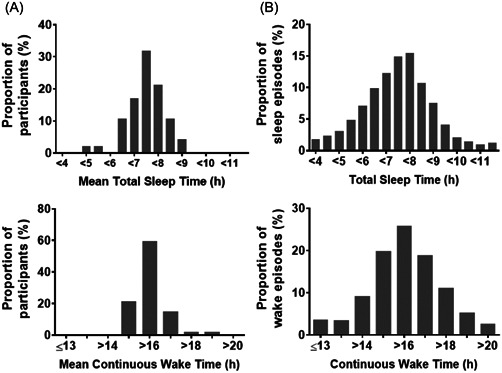
(A) Distribution of average subjective total sleep time (upper panel) and duration of continuous time awake per participant (lower panel) (*n* = 47). (B) Distribution of subjective total sleep time (upper panel, *n* = 6519) and time awake (lower panel, *n* = 6240) for all sleep episodes reported by expeditioners during the winter season.

The median number of nights contributed to total sleep time analyses by each participant (*n* = 47) was 158 nights (range: 27–210). The average subjective sleep duration per individual was 7.20 ± 0.78 h, with nearly a third (31.9%) of expeditioners obtaining < 7 h sleep per episode on average. When individual sleep episodes were examined, the mean subjective sleep duration for all episodes across the season was 7.22 ± 1.53 h. The incidence of sleep episodes shorter than 7, 6.5, and 6 h were 41.4% (2697/6519), 29.1% (1894/6519), and 19.2% (1249/6519), respectively (Figure 1).

The median number of wake episodes examined for each participant was 149 (range: 24–209). The mean duration of time awake between major sleep episodes per individual was 16.39 ± 0.78 h with most expeditioners (78.8%) staying awake longer than 16 h per day, on average. When individual wake episodes were assessed, the mean duration of time awake was 16.52 ± 1.92 h. The incidence of wake episodes longer than 16 h was 63.8% (3983/6240) and episodes longer than 18 h was 19.1% (1190/6240) (Figure 1).

**Figure 2:**
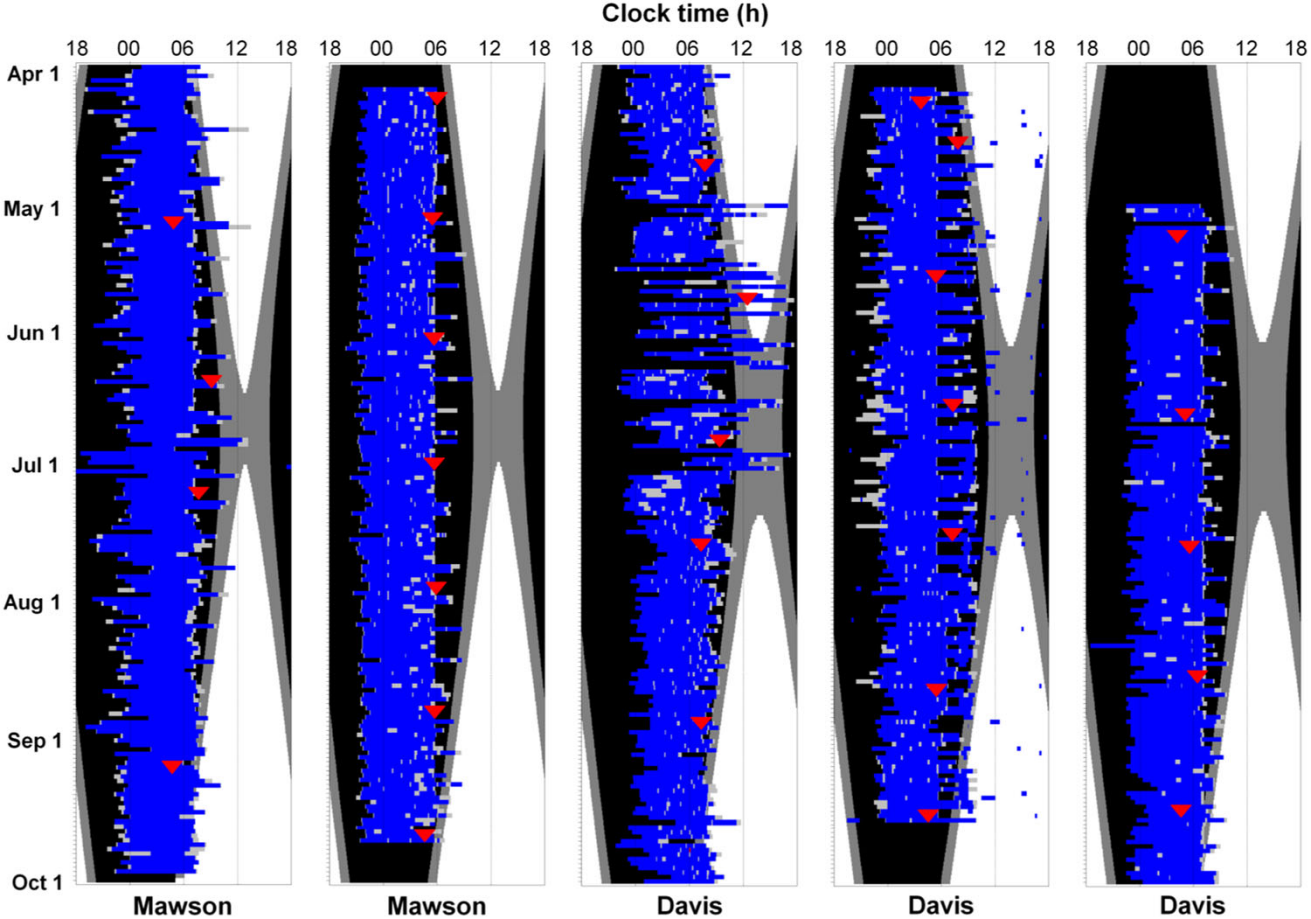
Raster plots of five example expeditioners demonstrating the variability in self‐reported sleep timing and duration (blue bars). Light gray bars represent sleep onset latency and awakenings after sleep onset. Acrophase each month (red triangles) was determined by cosinor analysis of aMT6s concentration (μg/h) on 48‐h urine collections. Black illustrates nautical twilight; dark gray illustrates civil twilight.

The average time of sleep onset during winter was 23:39 ± 1:45 h and the average time of waking was 7:05 ± 1:47 h. Considerable variability in the timing of sleep onset and offset was evident, however, with sleep onset and offset times covering most of the 24‐h day. One‐third of sleep episodes (33.6%; 2188/6522) began after midnight. Variability in sleep onset and offset time was evident both between and particularly within individuals. The majority of individuals displayed considerable variability in sleep timing across the season and often night‐to‐night. Examples of the variability in sleep duration and timing across the winter season are illustrated in Figure 2.

### 3.2 Circadian phase

**Figure 3:**
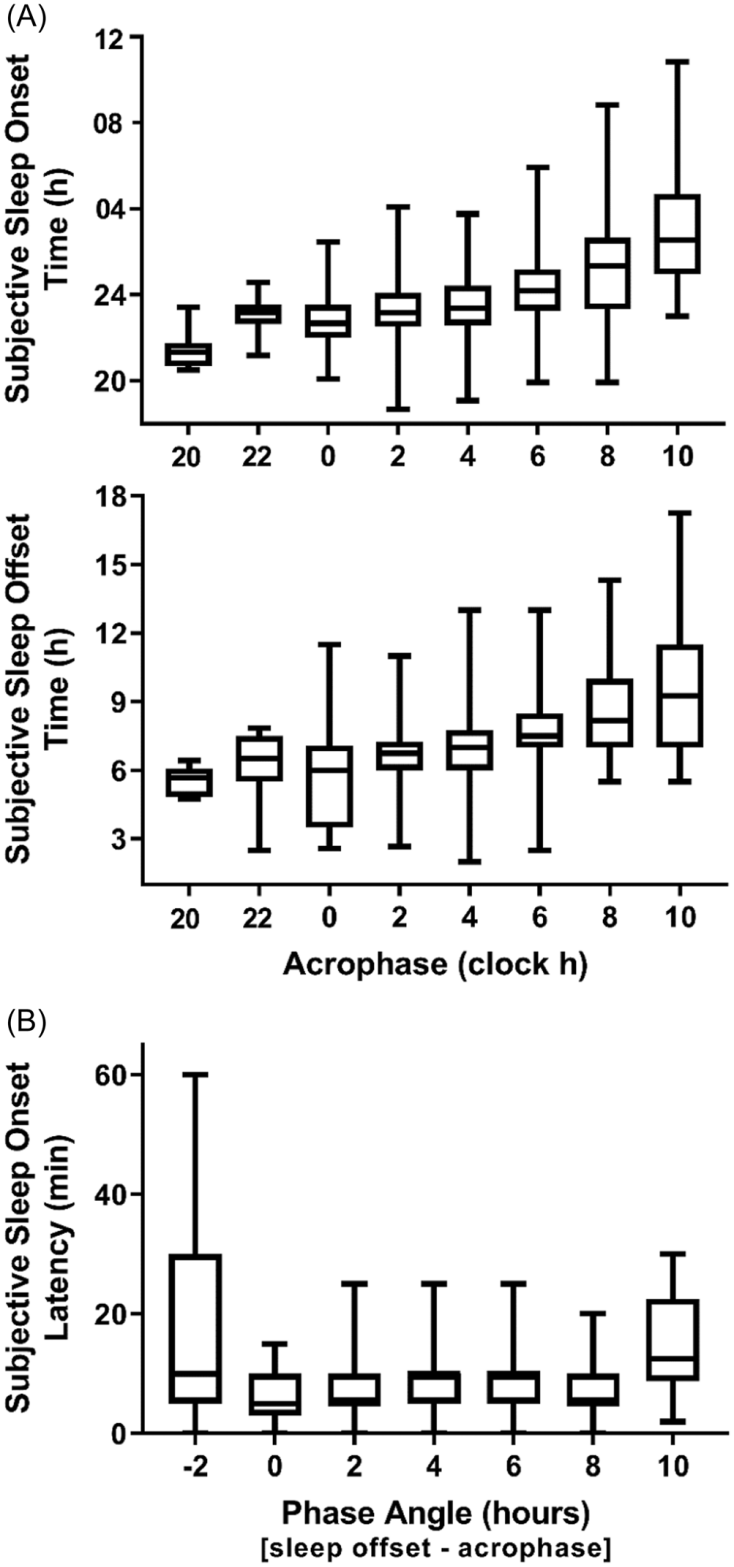
Subjective sleep measures in 44 expeditioners relative to circadian phase represented by (A) urinary aMT6s acrophase and (B) phase angle difference between aMT6s acrophase and subjective sleep offset (e.g., −2 represents an aMT6s acrophase occurring 2 h after sleep offset). Each boxplot presents the interquartile range as a box, with the median value presented with the horizontal line within the box. Whiskers represent the minimum and maximum values. Outliers identified by Q > 99% confidence limit have been removed.

The average time of all measured aMT6s acrophases across the winter was 4:50 ± 1:59 h, ranging from 22:31 to 11:54 h (*n* = 247). Considerable variability in the phase angle between acrophase (measured and interpolated) and subjective sleep offset was demonstrated with a range in phase angle of ‐10:44–8:21h (aMT6s rhythm peak ranging from 10.73 h before wake time to 8.35 h after wake). Figure 3 shows the relationship between circadian phase (acrophase and phase angle between acrophase and sleep offset) and subjective sleep timing, and sleep onset latency. Subjective sleep onset time and offset time were delayed with the later timing of acrophase (Figure 3A). Subjective sleep onset latency was longer when the phase angle was −2 h (acrophase 2 h after sleep offset) or 10 h (acrophase 10 h before sleep offset) compared with phase angles of 0–8 h (Figure 3B).

**Figure 4:**
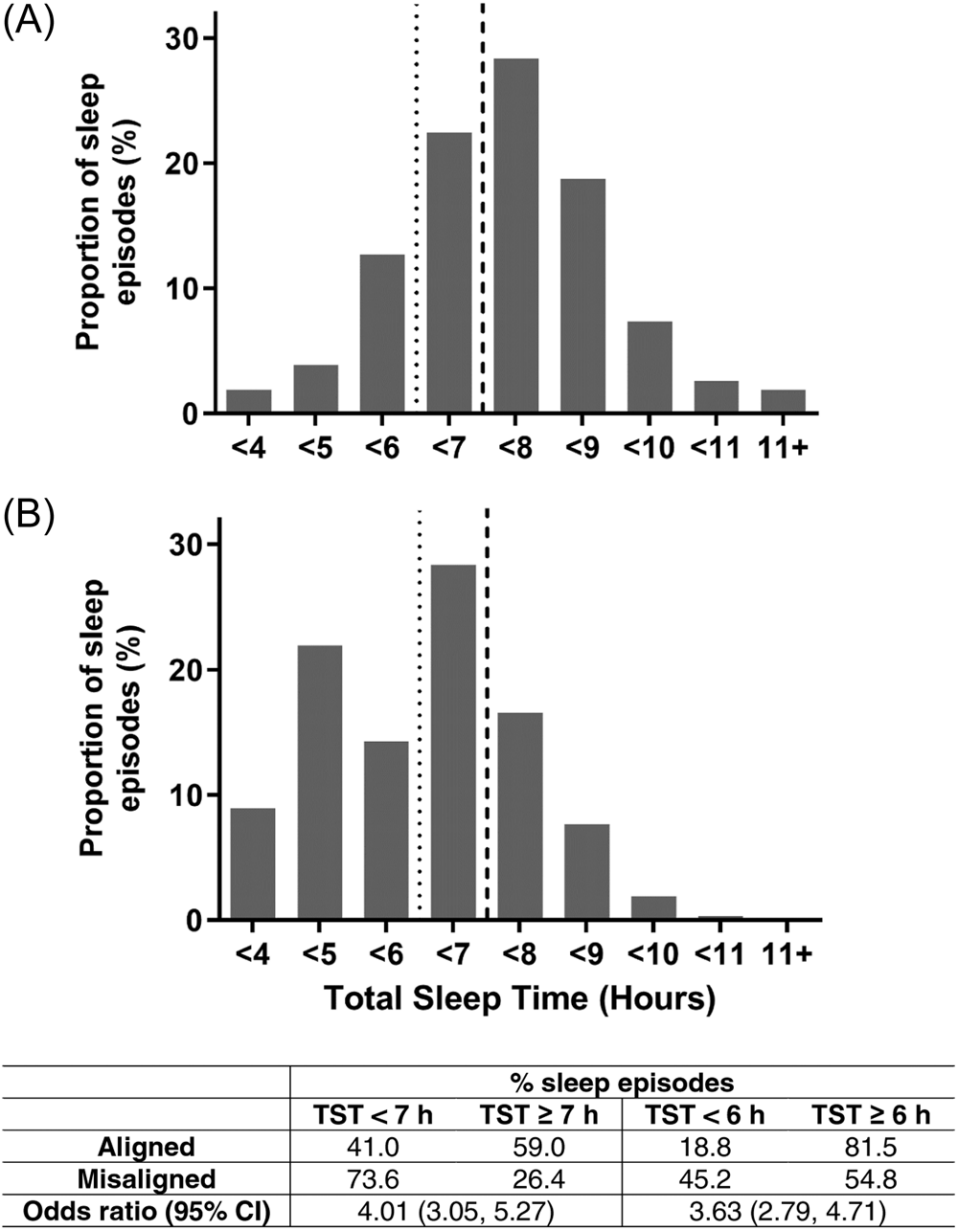
Subjective total sleep time during (A) aligned sleep episodes (aMT6s acrophase during sleep) (*n* = 1359) and (B) misaligned sleep episodes (aMT6s acrophase outside of sleep episode) (*n* = 314) for 34 expeditioners with both aligned and misaligned sleep. Dashed lines separate sleep episodes with subjective total sleep time shorter than 7 h (thick line) and 6 h (thin line). The table presents the proportion of aligned and misaligned sleep episodes associated with 6 and 7 h of sleep and the odds of obtaining <6 and 7 h of sleep when misaligned.

Misalignment of sleep from the circadian phase was defined as when the aMT6s acrophase occurred outside of the sleep episode. Of all sleep episodes during the season, the aMT6s acrophase occurred outside of the sleep episode (defined as misaligned) on 14.1% (314/2221) of occasions. Thirty‐four out of 50 expeditioners had some of their sleep episodes occur at an abnormal circadian phase, accounting for 18.8% (314/1673) of sleep episodes for these expeditioners. When the sleep episode was misaligned, expeditioners obtained significantly less sleep on average (6.14 ± 1.34 h), compared with when sleep was aligned (7.32 ± 0.96 h), (*t*(33) = 4.970, *p* < .0001). Of misaligned sleep episodes, only 26.4% were of at least 7 h duration, compared with 59.0% of aligned sleep episodes (Figure 4). Sleep duration was <6 h for 45.2% of misaligned sleep, compared with 18.8% <6 h for aligned sleep episodes (Figure 4). When a sleep episode was misaligned, the odds of obtaining less than 6 h of sleep was 3.6 (95% confidence interval [CI]: 2.8, 4.7), and obtaining 7 h of sleep was 4.0 (95% CI 3.1, 5.3), compared with when aligned (Figure 4). Expeditioners reported more waking after sleep onset when sleep episodes were misaligned (12.42 ± 12.78 min) compared with when aligned (7.15 ± 10.65 min), (*t*(33) = 3.523, *p* = .001).

### 3.3 Cognitive assessments

**Figure 5:**
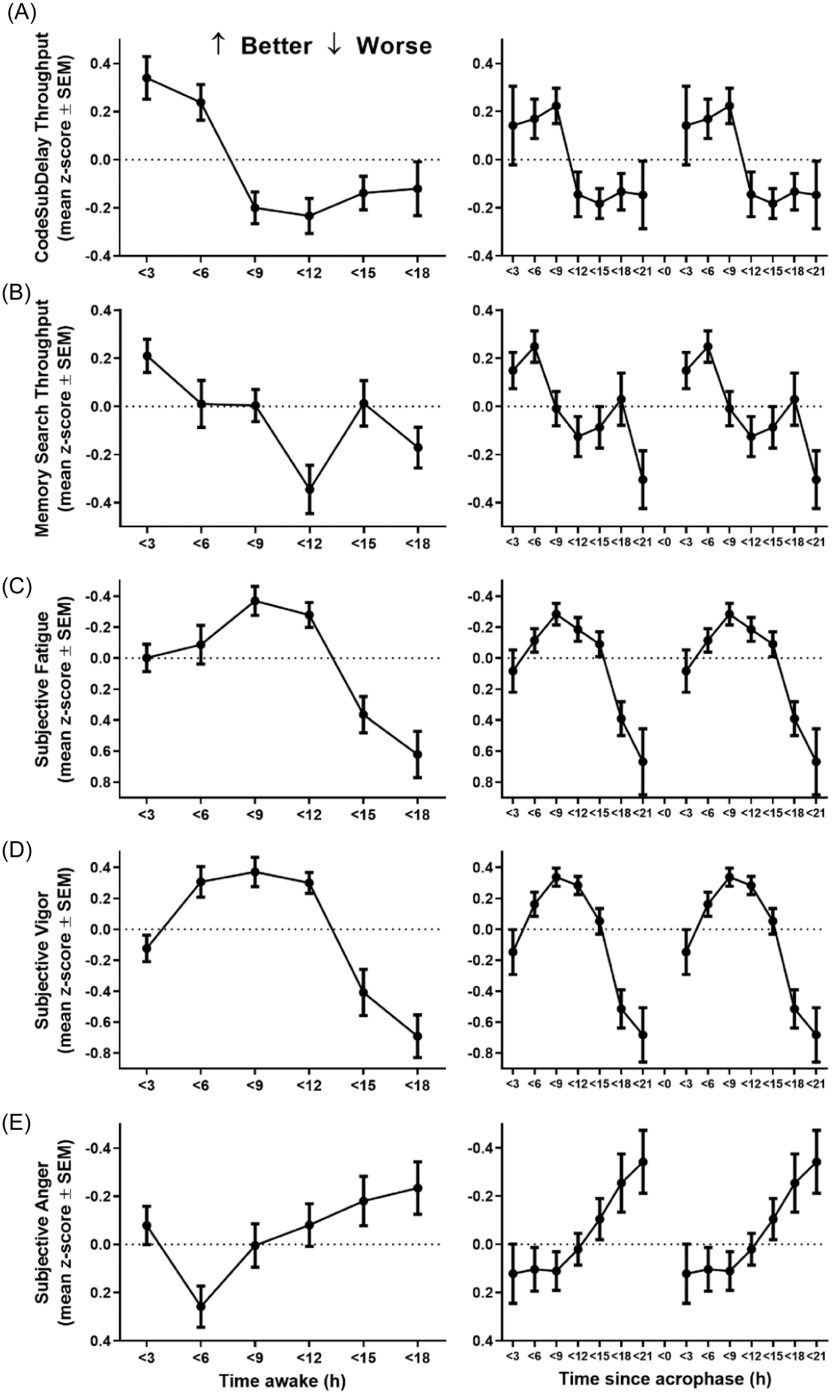
Performance and subjective mood were dependent on the number of continuous hours awake and circadian phase. Mean ± SEM (A) Code Substitution Delay throughput, (B) Memory Search throughput, (C) subjective fatigue, (D) subjective vigor, and (E) subjective anger with hours of time awake (left panels) and time since acrophase (right panels). Time since acrophase is double plotted.

Performance on ANAM‐ICE Code Substitution Delay and Memory Search worsened with increased time awake (*F*_5,130_ = 10.46, *p* < .0001 and *F*_5,138_ = 4.88, *p* < .001, respectively) and when assessed during the biological night (*F*_6,143_ = 4.55, *p* < .001 and *F*_6,154_ = 4.45, *p* < .001, respectively; Figure 5). Subjective ratings of fatigue (*F*_5,128_ = 13.29, *p* < .0001) and vigor (*F*_5,134_ = 17.64, *p* < .0001) were also poorer with increased time awake (Figure 5). Self‐reported fatigue was greater (*F*_6,135_ = 8.86, *p* < .0001) and vigor lower (*F*_6,143_ = 13.54, *p* < .0001) when assessed during the biological night (Figure 5). Anger diminished with time awake (*F*_5,122_ = 3.52, *p* < .0052) and time since acrophase (*F*_6,132_ = 3.19, *p* < .01).

**Figure 6:**
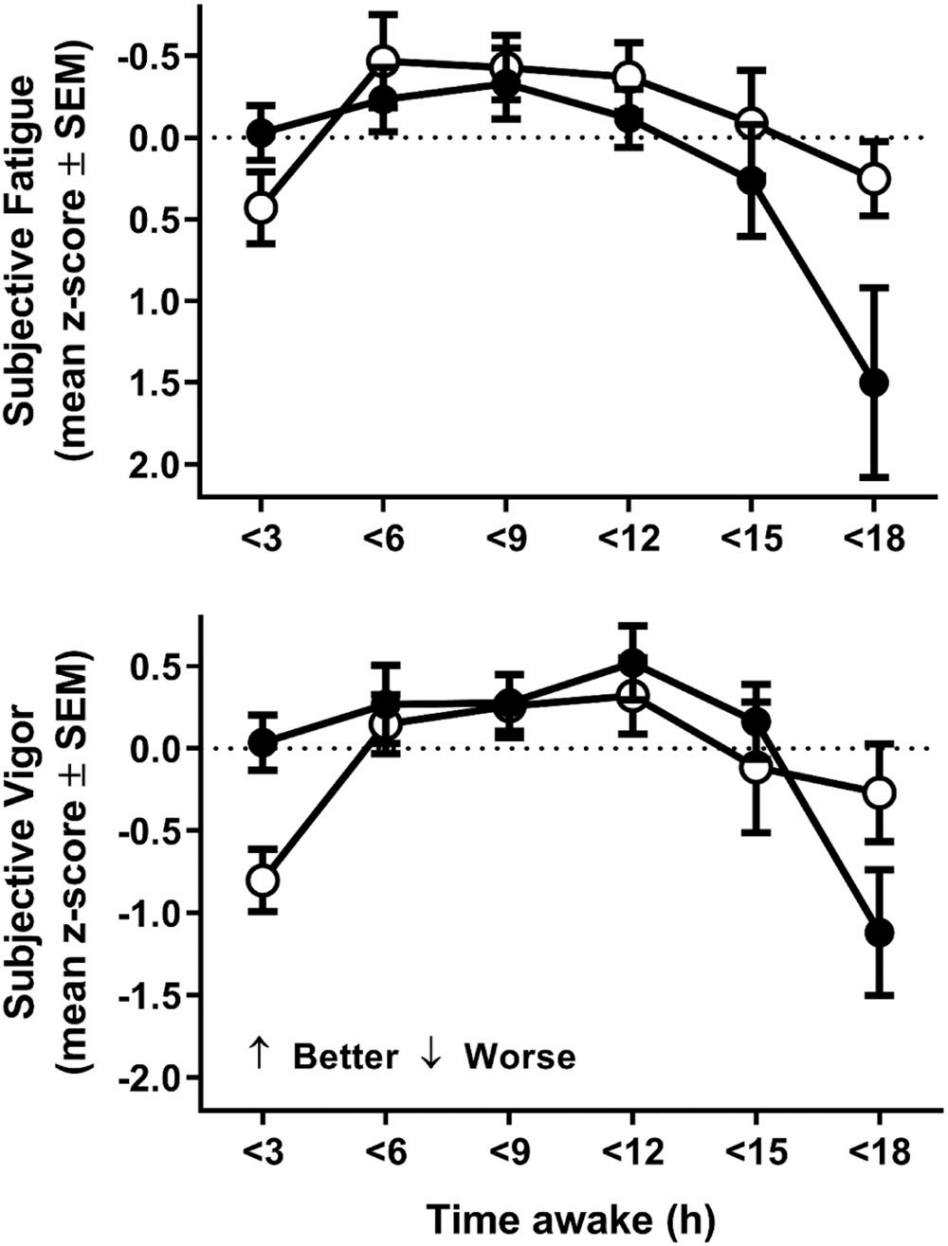
Average subjective fatigue and subjective vigor (mean *z*‐score ± SEM) plotted in relation to continuous time awake for expeditioners (*n* = 13) with tests on days following abnormally advanced acrophase relative to sleep, closed circles (phase angle at sleep offset 3.1 to 6.8 h, *n* = 102 tests) and abnormally delayed acrophase relative to sleep, open circles (phase angle at sleep offset 0.4 h to −7.9 h, *n* = 84 tests). Advanced and delayed acrophase was defined by the 25% most advanced and delayed acrophase relative to sleep (phase angle of entrainment). Note reduced “morning” sleepiness and higher vigor in advanced individuals waking at a later circadian phase, compared with delayed individuals waking at an earlier circadian phase.

Self‐reported fatigue (Advanced: *F*_5,69.3_ = 5.47, *p* < .001; Delayed: *F*_5,54.1_ = 3.89, *p* < .01) and vigor (Advanced: *F*_5,69.8_ = 5.19, *p* < .001; Delayed: *F*_5,53.2_ = 4.94, *p* < .001) differed with time awake on days of advanced and delayed phase (Figure 6). Profiles for days when the acrophase was abnormally delayed relative to sleep (25% most delayed relative to sleep), causing individuals to wake at an earlier circadian phase, were characterized by maximal fatigue and reduced vigor early in the wake episode followed by a general leveling for the following 15 h (Figure 6, open circles). Assessments on days when the acrophase was advanced relative to sleep (25% most advanced relative to sleep) such that individuals wake later in their circadian cycle, were associated with less fatigue and higher vigor earlier in the day, with a substantial decline toward the end of the wake episode (Figure 6, closed circles).

### 3.4 Psychological health assessments

Linear mixed models showed no change across season for scores for the Depression Anxiety Stress Scale (anxiety or stress), Positive or Negative Affect Schedule, or Patient Health Questionnaire (Table 2). Profile of Mood States‐Bi (Bi‐POMS) differed with time into season for subscales of tension‐anxiety (*F*_5,190_ = 2.77, *p* < .05), anger‐hostility (*F*_5,189_ = 3.66, *p* < .01), and fatigue‐inertia (*F*_5,189_ = 2.69, *p* < .05). Tension‐anxiety and anger‐hostility increased after the second month of the winter. Fatigue‐inertia symptoms peaked in June and July but remained high in September compared with April. Team member cohesiveness decreased across the winter (*F*_5,188_ = 3.36, *p* < .01) reaching a trough in September. Expeditioners were less likely to report at least moderate cohesiveness in September (58.3%) compared with April (96.3%) (*p* < .005). Team member exchange was not influenced by time across winter. Perception of relationship conflicts (*F*_5,189_ = 2.71, *p* < .05) and workstyle conflicts (*F*_5,189_ = 3.55, *p* < .005) increased during the winter, both with a peak in September. Less than one‐third (29.6%) of expeditioners identified at least a moderate degree of relationship conflict in the first month of winter compared with 73.0% in month 6 (*p* < .005). At least a moderate level of workstyle conflict was identified by 54.1% of expeditioners at the end of winter compared with 11.1% at the start of the season (*p* < .0001).

**Table 2:** Monthly survey assessments of psychological health, personality, diurnal preference, and team interactions

	April			May			June			July			August			September		
	*n*	Mean (SD)	%	*n*	Mean (SD)	%	*n*	Mean (SD)	%	*n*	Mean (SD)	%	*n*	Mean (SD)	%	*n*	Mean (SD)	%
Depression Anxiety Stress Scale																		
Anxiety^a^	27	1.04 (1.60)	0	28	1.00 (1.28)	0	32	1.94 (2.56)	6	30	1.33 (2.25)	3	31	1.42 (2.69)	3	35	1.54 (2.91)	6
Stress^b^	27	2.96 (2.79)	0	26	3.23 (3.76)	0	31	4.71 (4.34)	0	34	3.76 (4.74)	3	32	5.00 (5.66)	6	36	5.50 (5.68)	6
Positive and Negative Affect																		
Positive affect	26	35.58 (5.87)		26	35.69 (6.33)		31	33.23 (6.58)		33	34.67 (7.04)		31	33.16 (7.02)		35	33.69 (7.15)	
Negative affect	21	10.86 (1.20)		25	10.92 (1.89)		28	12.64 (5.33)		31	12.26 (4.63)		31	11.90 (3.52)		31	12.26 (4.12)	
Patient Health Questionnaire^c^	25	11.36 (2.04)	4	27	10.74 (1.48)	4	31	11.90 (2.97)	16	32	12.22 (2.77)	19	32	11.97 (2.85)	22	36	12.19 (3.17)	22
Homicidal ideation^d^	27	1.00 (0.00)	0	29	1.03 (0.19)	3	33	1.03 (0.17)	3	35	1.00 (0.00)	0	35	1.00 (0.00)	0	36	1.03 (0.17)	3
Degree of difficulty^e^	23	1.09 (0.29)	9	27	1.04 (0.19)	4	30	1.27 (0.45)	27	33	1.24 (0.50)	21	30	1.20 (0.41)	20	33	1.12 (0.33)	12
Profile of Mood States																		
Total mood disturbance	25	13.84 (9.53)		28	17.36 (9.01)		31	22.00 (17.66)		33	22.85 (14.96)		34	21.50 (13.10)		35	20.86 (12.91)	
Tension‐anxiety	27	4.37 (2.39)		29	5.14 (0.35)		33	6.15 (3.37)		35	5.91 (2.48)		35	5.83 (1.90)		37	6.08 (1.82)	
Depression‐dejection	27	4.96 (2.07)		29	6.07 (1.98)		33	6.85 (3.66)		35	6.74 (3.09)		35	6.69 (2.53)		37	6.76 (2.89)	
Anger‐hostility	27	4.37 (2.44)		29	5.17 (0.47)		33	5.91 (2.44)		34	6.18 (2.47)		35	6.17 (1.89)		37	6.14 (2.04)	
Vigor‐activity	27	15.00 (3.88)		29	14.90 (4.77)		33	14.39 (4.94)		35	14.09 (5.45)		35	14.20 (5.35)		36	15.42 (5.00)	
Fatigue‐inertia	27	6.63 (2.92)		29	7.79 (3.20)		33	9.55 (4.83)		34	9.68 (3.98)		35	9.26 (4.58)		37	9.08 (3.62)	
Confusion‐bewilderment	27	7.26 (2.68)		29	7.86 (0.99)		33	8.21 (2.06)		35	7.94 (1.71)		35	7.66 (1.03)		37	8.05 (1.49)	
BHP TME																		
Cohesiveness^f^	27	4.49 (0.43)	96	28	4.14 (0.71)	79	33	3.98 (0.61)	67	35	4.04 (0.80)	74	35	3.96 (0.77)	60	36	3.78 (0.84)	58
Exchange^f^	27	3.77 (0.38)	37	28	3.71 (0.48)	32	33	3.68 (0.41)	27	35	3.62 (0.59)	20	35	3.74 (0.48)	37	36	3.68 (0.50)	19
Perception of Current Conflicts																		
Relationship^g^	27	2.12 (1.06)	30	28	2.52 (1.19)	50	33	2.89 (1.20)	70	35	2.65 (1.17)	60	35	3.02 (1.40)	71	37	3.16 (1.48)	73
Workstyle^g^	27	1.51 (0.54)	11	28	1.78 (0.64)	25	33	1.80 (0.63)	27	35	1.87 (0.80)	26	35	2.15 (0.94)	49	37	2.22 (0.96)	54
Morningness‐Eveningness^h^	23	53.61 (9.67)	35	8	59.75 (11.99)	50										26	55.92 (9.81)	54
BHP Personality Questionnaire																		
Total score	20	175.40 (12.33)		8	182.75 (21.68)											16	173.81 (12.81)	
Agreeableness	27	4.22 (0.39)		8	4.53 (0.35)											25	4.09 (0.62)	
Conscientiousness	27	4.11 (0.52)		8	4.26 (0.51)											25	4.09 (0.52)	
Emotional stability	27	4.26 (0.48)		8	4.50 (0.47)											25	4.04 (0.63)	
Extraversion	27	3.09 (0.73)		8	3.41 (1.13)											25	2.99 (0.63)	
Openness to experience	27	3.84 (0.53)		8	3.84 (0.66)											25	3.78 (0.51)	

*Note*: % = % above threshold.

^a^ DASS Anxiety at least mild (score ≥ 8).

^b^ DASS Stress at least mild (score ≥ 15).

^c^ PHQ at least moderately severe depression (score ≥ 15).

^d^ PHQ homicidal ideation % individuals reporting thoughts of hurting or killing someone else.

^e^ PHQ degree of impairment very – extremely difficult (score ≥ 2).

^f^ Cohesiveness and team exchange scores ≥4 (moderately & very accurate).

^g^ Perception of Current Conflicts >2 (moderate or high conflict).

^h^ MEQ score ≥59 (morning type).

For subset analyses based on time on station, individuals who had been on station for both the summer and winter season reported worse scores for Positive Affect (*F*_1,53_ = 7.20, *p* < .05), Patient Health Questionnaire (*F*_1,53_ = 8.00, *p* < .01), Bi‐POMS tension‐anxiety (*F*_1,58_ = 4.21, *p* < .05), Bi‐POMS fatigue‐inertia (*F*_1,58_ = 6.82, *p* < .05), Bi‐POMS vigor‐activity (*F*_1,57_ = 4.58, *p* < .05), and total mood disturbance (*F*_1,54_ = 4.72, *p* < .05), compared with expeditioners arriving at the start of the winter (Supporting Information: Figure 2). Compared with the start of winter, assessments late in winter revealed worse scores for Positive Affect (*F*_1,53_ = 4.41, *p* < .05), Patient Health Questionnaire (*F*_1,53_ = 5.16, *p* < .05), DASS Stress (*F*_1,56_ = 4.66, *p* < .05), Bi‐POMS depression‐dejection (*F*_1,58_ = 4.41, *p* < .05), and Bi‐POMS anger‐hostility (*F*_1,58_ = 6.16, *p* < .05).

## 4 DISCUSSION

In expeditioners over‐wintering in Antarctica, misalignment between the circadian pacemaker relative to the timing of sleep was prevalent, occurring on almost one‐fifth of sleep episodes. It was common for expeditioners to obtain less than 7 h of sleep. When sleep was misaligned with the circadian phase, expeditioners obtained over 1 h less sleep compared with when sleep episodes occurred during the biological night. Waking performance and self‐reported fatigue and vigor varied based on time awake and circadian phase in a predictable manner. The temporal profile of self‐reported fatigue and vigor with time awake was also dependent on circadian phase. Evidence of deterioration of mood and team cohesion was observed across the winter expedition but the incidence of symptoms reaching clinical significance did not change.

Comparing the mean sleep duration per participant showed that one‐third (32%) of participants obtained less than 7 h of sleep on average, a rate similar to reports of average sleep duration obtained by adults in the United States (35%).^53^ This study extends previous reports of short sleep averaged over time in the Antarctic environment^6^, ^7^, ^10^, ^18^ by examining the distribution of short sleep episodes. Perhaps more important than mean sleep duration is the frequency of short sleep episodes. When sleep duration was assessed by episode, the incidence of sleep shorter than 7 h increased to 41%, demonstrating that assessment of mean sleep duration often provides an underestimate of sleep deficit and substantial inter‐ and intra‐individual variation exists. The recommended adult sleep duration for optimal health is 7 or more hours with sleeping less than 7 h increasing the risk of poor quality of life, weight gain, diabetes, cardiovascular disorders, reduced immune response, depression, and mortality.^54^ Optimizing the sleep obtained in the Antarctic environment is therefore important for expeditioner health, especially during such long‐duration missions.

Sleep duration was shorter when the timing of sleep was misaligned with the aMT6s peak. This finding of reduced sleep with circadian misalignment extends previous reports of changes in sleep with circadian phase in healthy adults in the laboratory,^15^, ^55^, ^56^ in blind individuals^57^ and in operational settings with altered light‐dark cycles.^11^, ^41^, ^52^ When misalignment between sleep and the circadian pacemaker had expeditioners attempting sleep during the wake maintenance zone (acrophase occurring 2 h after waking indicating individuals are attempting sleep at an earlier biological time, Figure 3), they experienced extended sleep onset latency, a pattern that is also observed in blind individuals sleeping at an inappropriate circadian phase.^58^

Considerable variability was observed in circadian timing, as measured by the peak of the aMT6s rhythm (17‐h range), leading to substantial variation in the circadian timing of sleep. Two‐thirds of expeditioners experienced some misalignment between the timing of sleep and the circadian pacemaker during the expedition. Sleep episodes were misaligned with circadian phase on one‐fifth of occasions. Circadian misalignment in some expeditioners may be due to endogenous differences in circadian timing^32^, ^33^ or imposition of duty requiring abnormal timing of sleep. As illustrated in Figure 2, for example, expeditioners were often required to wake early due to duty obligations compared with a considerably later wake time on rest days. Altered ocular light exposure, the primary time cue for circadian entrainment,^1^ is likely to play a predominant role in circadian timing during the Antarctic winter. Antarctic winters are associated with a delay or free running of the melatonin rhythm^8^, ^13^ while the timing of sleep is unchanged, thereby resulting in circadian misalignment. The reason for the delay is unclear, however, as although outdoor light levels are diminished, indoor station lighting can be maintained at standard levels (~300–600 lux)^11^, ^40^ that are likely to be sufficient for circadian entrainment,^59^, ^60^ albeit sometimes at a suboptimal phase angle.^11^ Increased sensitivity to light exposure during the winter^61^, ^62^ could theoretically assist with entrainment of the circadian pacemaker. Despite the presence of electric lighting on stations, however, the incidence of circadian misalignment across the season and between individual expeditioners remained high, suggesting that electric lighting could be improved. The incidence of circadian misalignment in the Antarctic environment is very similar to rates observed in simulated and actual space missions,^37^, ^41^, ^52^ reinforcing the fidelity of Antarctica as a space flight analog and the need to address this common problem. In patients with insomnia, the proportion of individuals initiating sleep at too early a circadian phase is 10%–22% depending on the definition used.^63^ Our finding that 19% of sleep episodes were at an adverse circadian phase suggests a high prevalence consistent with other populations exhibiting sleep and circadian disorders.

In this real‐world Antarctic study, circadian misalignment was defined using the broad definition of the aMT6s acrophase occurring outside of the sleep episode (and similarly when temperature minimum was estimated to occur outside of sleep in the ISS study^52^). It is noted that misalignment may also be present when the acrophase occurs within the sleep episode but does not occur at the optimal time relative to the sleep episode to be conducive to sufficient duration and quality of sleep. Advanced or delayed circadian timing within the sleep episode can be associated with considerable sleep disruption^14^ and therefore the incidence of circadian misalignment reported is likely to be an underestimate.

The effects of circadian misalignment on sleep loss, performance, and mood have considerable immediate safety implications, and potentially longer term health risks across multiple systems including immune, metabolic, cardiovascular, and psychological functions.^2^, ^3^, ^64^, ^65^ Circadian misalignment and associated sleep disturbances and/or inappropriate meal timing, for example, are associated with impaired glucose tolerance, high blood pressure, increased risk of diabetes, and increased inflammatory markers.^3^, ^65^ In the short term, the safety of an Antarctic expedition in extreme winter conditions of darkness, cold and poor visibility depends on the ability of expeditioners to remain alert. The average amount of sleep obtained during the winter, particularly during circadian misalignment is below the amount required to avoid impairments in cognitive performance^66^, ^67^ and the risk of accidents and injuries.^68^, ^69^ Performance and subjective ratings of fatigue and vigor were dependent on sleep and circadian regulation principles. Expeditioners demonstrated more impairment with increasing time awake and when performing during their biological night, as found under controlled laboratory settings.^70^ Subjective ratings also demonstrated evidence of sleep inertia with initial improvement in the hours after waking before subsequent deterioration, as identified in laboratory settings.^70^

Despite the variability in factors associated with the field environment, including motivation, training, and use of stimulants such as caffeine and light, differences in the relationship between circadian entrainment and the time course of subjective fatigue ratings were also evident, albeit in a smaller sample size (*n* = 13). This evidence replicates findings from other field studies,^57^ and reflects endogenous differences in phase angle of entrainment between morning and evening types.^71^ Individuals who woke at a later phase of the circadian cycle demonstrated an earlier peak in alertness followed by a decline across the day compared with more delayed individuals who woke at an earlier phase and experienced poorer alertness in the morning.^57^ These findings demonstrate the need to ensure appropriate alignment of circadian phase in future Antarctic expeditions and long‐duration space flights to optimize sleep and expeditioner safety.

Symptoms of depressed mood, tension, and fatigue increased across the season, along with increased interpersonal tension and conflict toward team members, as seen previously.^21^, ^22^, ^23^, ^72^ The pattern of mood changes was more closely associated with increased time into expedition than photoperiod changes across the season and may be associated with confinement and other behavioral factors.^23^ There was no evidence in our study of the inconsistent third quarter phenomenon^24^, ^25^ either for changes in mood outcomes across the winter season or in the subset assessment of time on station, where the end of the participation period reflects the end of the expedition.

The current data demonstrating the relatively high incidence of circadian misalignment during Antarctic expeditions in the winter should inform the optimization of operational countermeasures during future long‐duration missions, particularly for individuals most at risk of circadian desynchrony and sleep disruption. For example, properly planned and controlled lighting interventions with the appropriate light level, spectrum, and timing could be applied to entrain the circadian pacemaker and to improve the quality and duration of sleep during Antarctic missions and long‐duration space missions. Provision of a 24‐h light‐dark cycle in an operational environment does not guarantee entrainment^13^, ^37^ nor consistent and appropriate circadian alignment as this study and others^52^ have shown. Dynamic lighting with appropriate timing of light and dark may include enhanced light exposure in the morning to advance the circadian phase in individuals at risk of delays in the absence of natural morning light. Previous lighting changes at Antarctic stations have demonstrated that exposure to extra bright white light in both the morning and evening,^8^ in the morning^40^, ^73^ or both bright white and blue‐enriched light throughout the day^6^, ^10^ counteracts circadian phase delay in expeditioners, along with earlier sleep timing^6^, ^40^ and enhanced wellbeing, alertness, and mood.^10^, ^73^ The British Antarctic Survey Base of Halley 6, for example, has been equipped with a lighting system designed to maintain circadian synchrony in the Polar winter and to improve mood and performance (Martin Valentine, architect). Development of tools so that expeditioners could gain knowledge of their circadian timing may allow them to better align sleep episodes to times of peak sleep propensity.^74^

Limitations of the current study include a lack of assessments before departure from mainland Australia. Extrapolation of the timing of circadian acrophase between monthly assessments assumed a straight line between measurements rather than free‐run or scalloping of the circadian pacemaker. Participants are unlikely to have free‐run between monthly assessments as they would have required a large shift of 24‐h in 28 days and a long period length, of which there is no evidence in the sleep data. Although extrapolation was limited to 5 days, future studies may consider more frequent measurement of circadian phase. Future research should examine the impact of individual light exposure on circadian misalignment in this environment, and conduct comparative assessments during the Austral summer which has also been associated with reduced sleep quality, cognitive performance, and delayed circadian timing,^28^, ^75^ most likely due to suboptimal provision of a sufficient dark episode. While some prior research has conducted direct comparisons between seasons and identified, for example, higher sleep fragmentation during summer compared with winter,^75^ the majority of research has examined average sleep, circadian or performance outcomes and has not examined the specific impacts of individual events of circadian misalignment. This study represents the largest and most comprehensive study conducted on circadian misalignment, sleep, performance, and psychological health of over‐wintering Antarctic expeditioners, conducted across multiple stations. The isolated conditions of the Antarctic environment with a highly changeable natural light cycle provide a unique analog for long‐duration space missions and the current study reconfirms the feasibility of assessing circadian phase and sleep behavior in extreme and remote environments. Expeditioners experience a high incidence of misalignment between the circadian pacemaker and timing of sleep, and obtain 1–2 h less sleep than the recommended amount, increasing the risk of performance impairments, compromised safety, and adverse health outcomes. Future research should examine the most appropriate interventions for circadian misalignment in this and other remote and dangerous environments.

## CONFLICTS OF INTEREST

Tracey L. Sletten reports her institution has received equipment donations or other support from Philips Lighting, Philips Respironics, Optalert™ and Compumedics. Dr. Sletten served as a Project Leader in the Cooperative Research Centre for Alertness, Safety, and Productivity. Laura K. Barger reports consulting fees from the University of Pittsburgh, CASIS, Puget Sound Pilots, the Centers for Disease Control and Prevention, Boston Children's Hospital, and Charles Czeisler. Shantha M.W. Rajaratnam reports that he has served as a consultant through his institution to Vanda Pharmaceuticals, Philips Respironics, EdanSafe, The Australian Workers' Union, National Transport Commission, and Transport Accident Commission, and has through his institution received research grants and/or unrestricted educational grants from Vanda Pharmaceuticals, Takeda Pharmaceuticals North America, Philips Lighting, Philips Respironics, Cephalon, and ResMed Foundation, and reimbursements for conference travel expenses from Vanda Pharmaceuticals. His institution has received equipment donations or other support from Optalert™, Compumedics, and Tyco Healthcare. He has also served as an expert witness and/or consultant to shift work organizations. Dr. Rajaratnam served as a Program Leader in the Cooperative Research Centre for Alertness, Safety, and Productivity. Jeff Ayton reports he serves as Chief Medical Officer and Program Lead for human biology and medicine research for the Australian Antarctic Program responsible for the health and medical care of participants in the study. Steven W. Lockley reports commercial interests from the last 3 years (2018‐2021). His interests are reviewed and managed by Brigham and Women's Hospital and Partners HealthCare in accordance with their conflict of interest policies. No interests are directly related to the research or topic reported in this paper but, in the interests of full disclosure, are outlined below. Steven W. Lockley has received consulting fees from BHP Billiton, EyeJust Inc., Noble Insights, Rec Room, Six Senses, Stantec, and Team C Racing; and has current consulting contracts with Akili Interactive; Apex 2100 Ltd.; Consumer Sleep Solutions; Headwaters Inc.; Hintsa Performance AG; KBR Wyle Services, Light Cognitive; Lighting Science Group corporation/HealthE; Mental Workout/Timeshifter and View Inc. He has received honoraria and travel or accommodation expenses from Bloxhub, Emory University, Estée Lauder, Ineos, MIT, Roxbury Latin School, and the University of Toronto, and travel or accommodation expenses (no honoraria) from IES, Mental Workout, Solemma, and Wiley; and royalties from Oxford University Press. He holds equity in iSleep pty. He has received an unrestricted equipment gift from F. Lux Software LLC, a fellowship gift from Stockgrand Ltd and holds an investigator‐initiated grant from F. Lux Software LLC, and a Clinical Research Support Agreement and Clinical Trial Agreement with Vanda Pharmaceuticals Inc. He is an unpaid Board Member of the Midwest Lighting Institute (non‐profit). He was a Program Leader for the CRC for Alertness, Safety and Productivity, Australia, through an adjunct professor position at Monash University (2015‐2019). He is a part‐time adjunct professor at the University of Surrey, UK. He holds patents for “Display screen or portion thereof with graphical user interface” (US USD897362S1, 2020, Published), “Method and system for generating and providing notifications for a circadian shift protocol” (US20190366032A1, pending), and “Method to shift circadian rhythm responsive to future therapy” (US 20210162164, 2021, pending). He has served as a paid expert in legal proceedings related to light, sleep, shiftwork, and health. The remaining authors declare no conflict of interest.

## Supporting information

Supporting information.

## Data Availability

The data that support the findings of this study are available on request from the corresponding author. The data are not publicly available due to privacy or ethical restrictions.
