# Supplementary material for: The role of circadian phase in sleep and performance during Antarctic winter expeditions
Source: J Pineal Res. 2022 Jul 25;73(2):e12817. doi: 10.1111/jpi.12817 (PMC9541096; doi:10.1111/jpi.12817)
Supplement: Supplementary file 1 — Supporting information. [file JPI-73-e12817-s001.docx]

**The role of circadian phase in sleep and performance during Antarctic winter expeditions.**

**Supporting Information**

**Materials and Methods**

*Objective sleep assessment*

Sleep was assessed with actigraphy in 23 expeditioners (n = 2566 sleep episodes). Mean actigraphic sleep duration was 6.47 ± 1.53 hours. Total sleep time was <7 hours on 63.1% (1619/2566) of sleep episodes and <6 hours for 30.1% (772/2566) of sleep episodes. Nineteen expeditioners (82%) wearing actiwatches had some of their sleep episodes occur at abnormal circadian phase. For these individuals, a total of 22% of sleep episodes (458/2104) were misaligned with aMT6s acrophase.

When the sleep episode was misaligned expeditioners obtained significantly less sleep on average (5.43 ± 1.23 h), compared to when sleep was aligned (6.62 ± 0.89 h), (t(18) = 6.541, p < 0.0001). Shorter sleep duration for misaligned sleeps was associated with shorter time in bed (7.42 ± 1.08 vs 8.34 ± 0.90, p = 0.003) and reduced sleep efficiency (73.38 ± 11.66 vs 79.80 ± 7.03, p = 0.003). Of misaligned sleep episodes, only 31.9% were at least 7 h duration, compared to 56.3% of aligned sleep episodes (Supplementary Figure 1). Sleep duration was <6 hours for 43.8% of misaligned sleeps, compared to 17.6% <6 h for aligned sleep episodes (Supplementary Figure 1). When a sleep episode was misaligned compared to aligned the odds of obtaining less than 6 hours of sleep was 3.0 and obtaining 7 hours sleep was 2.8 (Supplementary Figure 1).

*Cognitive assessments*

The Code Substitution Delayed Memory test is based on the digit symbol coding paradigm and is designed to assess sustained attention, working memory, and learning. In an initial learning trial, a set of nine symbols and nine digits were paired in a key on the upper portion of the screen and a symbol-digit test pair presented at the bottom of the screen. Participants indicated whether the test pair matched the pairing in the key. A delayed recall trial presented after other tasks provided no key and participants responded based on memory of the pairings during the learning trial. The Memory Search task assesses verbal working memory, immediate recognition and attention. Participants were presented with a series of 6 letters to memorize. The set was removed from view and individual letters were presented one at a time for the participant to indicate whether the probe letter matched any of the memory set letters. For both the Code Substitution Delay and Memory Search task, the outcome variable was throughput, calculated as the number of correct responses per minute.[^44^](#_ENREF_44)

ANAM-ICE sessions were completed on participants’ personal computers or on communal computers on station. ANAM-ICE data for participants who contributed to at least 10 tests across the season and had corresponding phase angle data were included in the analysis. These participants (n = 33) completed 1343 individual performance test batteries, with some loss of test battery data due to limiting work schedules or technical issues with software. The 33 participants completed at least 12 ANAM test batteries, the average number of tests per participant was 40.7 ± 16.4, out of a scheduled 42-56 per participant.

*Psychological health assessments*

The short (30-item) form of the Profile of Mood States (POMS-SF)[^47^](#_ENREF_47) was administered to assess mood on six sub-scales including tension-anxiety, depression-dejection, anger-hostility, vigor-activity, fatigue-inertia, and confusion-bewilderment, in addition to a score on Total Mood Disturbance (TMD) as a global measure of affective state. A series of Team Measures Questionnaires were implemented as compiled by the NASA Wyle Behavioral Health and Performance (BHP) Team. The Positive Affect Negative Affect Schedule (PANAS) psychometric scale measured constructs of positive and negative affect both as states and traits[^48^](#_ENREF_48) with higher values indicating more positive and negative affect, respectively. Symptoms of anxiety and stress were assessed with the Depression Anxiety Stress Scale (DASS).[^49^](#_ENREF_49) The Anxiety component consisted of seven items on dysphoria, hopelessness, devaluation of life, self-deprecation, lack of interest/involvement, anhedonia, and inertia. The Stress component consisted of seven items covering difficulty relaxing, nervous arousal, easily upset/agitation, irritability/over-reactivity, and impatience. The Depression component of the DASS was not administered. The Patient Health Questionnaire (PHQ), completed as an assessment of depression, contained nine symptom items from the Diagnostic and Statistical Manual of Mental Disorders-IV (DSM-IV),[^50^](#_ENREF_50) with higher total scores indicating greater depression symptom severity. In addition to the original nine questions, the administered version contained two additional questions to assess (1) the presence of thoughts of hurting or killing someone else, and (2) the level of difficulty to work and get along with others. At the beginning and end of the season, the BHP Personality Questionnaire, formed of items from the International Personality Item Pool (IPIP), examined **personality based on the Big Five characteristics: Agreeableness, Extraversion, Emotional Stability, Openness to Experience, and Conscientiousness.**

The Perception of Current Conflict (PCC) scale included two dimensions of intra-group conflict to examine relationship (personality) and workstyle (disruptive work behaviours) conflict.[^51^](#_ENREF_51) Higher values indicate greater degree of conflict. The quality of relationship between expeditioners and their team members (team member cohesion and exchange) were assessed with the BHP Team Member Exchange Questionnaire.[^52^](#_ENREF_52) For team cohesiveness, an average score was calculated from 3 statements related to team communication, togetherness and trust.


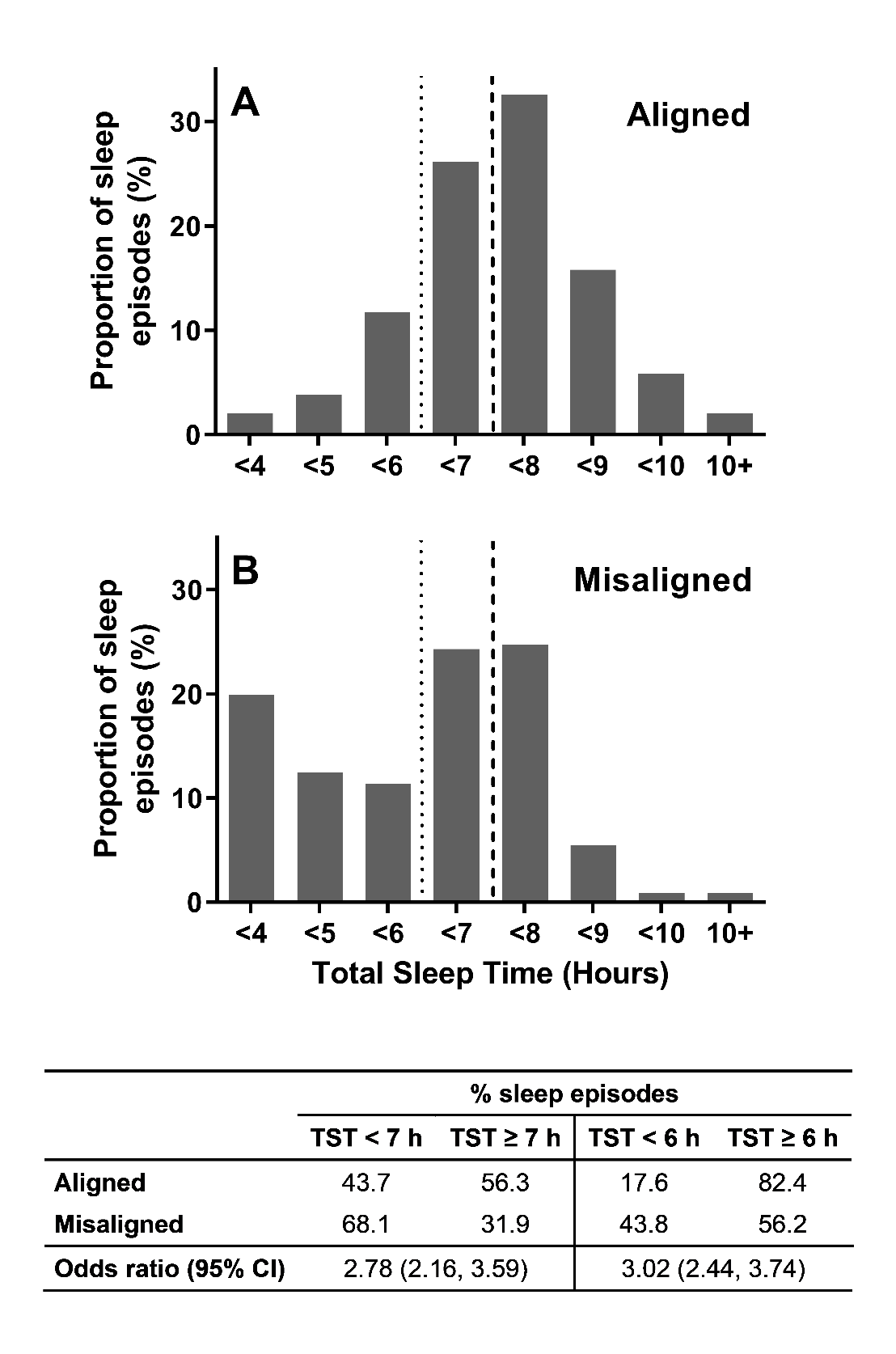


**Supplementary Figure 1.** Actigraphic total sleep time during (A) aligned sleep episodes (aMT6s acrophase during sleep) (n = 1646) and (B) misaligned sleep episodes (aMT6s acrophase outside of sleep episode) (n = 458) for 19 expeditioners with both aligned and misaligned sleeps. Dashed lines separate sleep episodes with subjective total sleep time shorter than 7 h (thick line) and 6 h (thin line). The table presents the proportion of aligned and misaligned sleep episodes associated with 6 and 7 h of sleep and the odds of obtaining <6 and 7 h of sleep when misaligned.

**
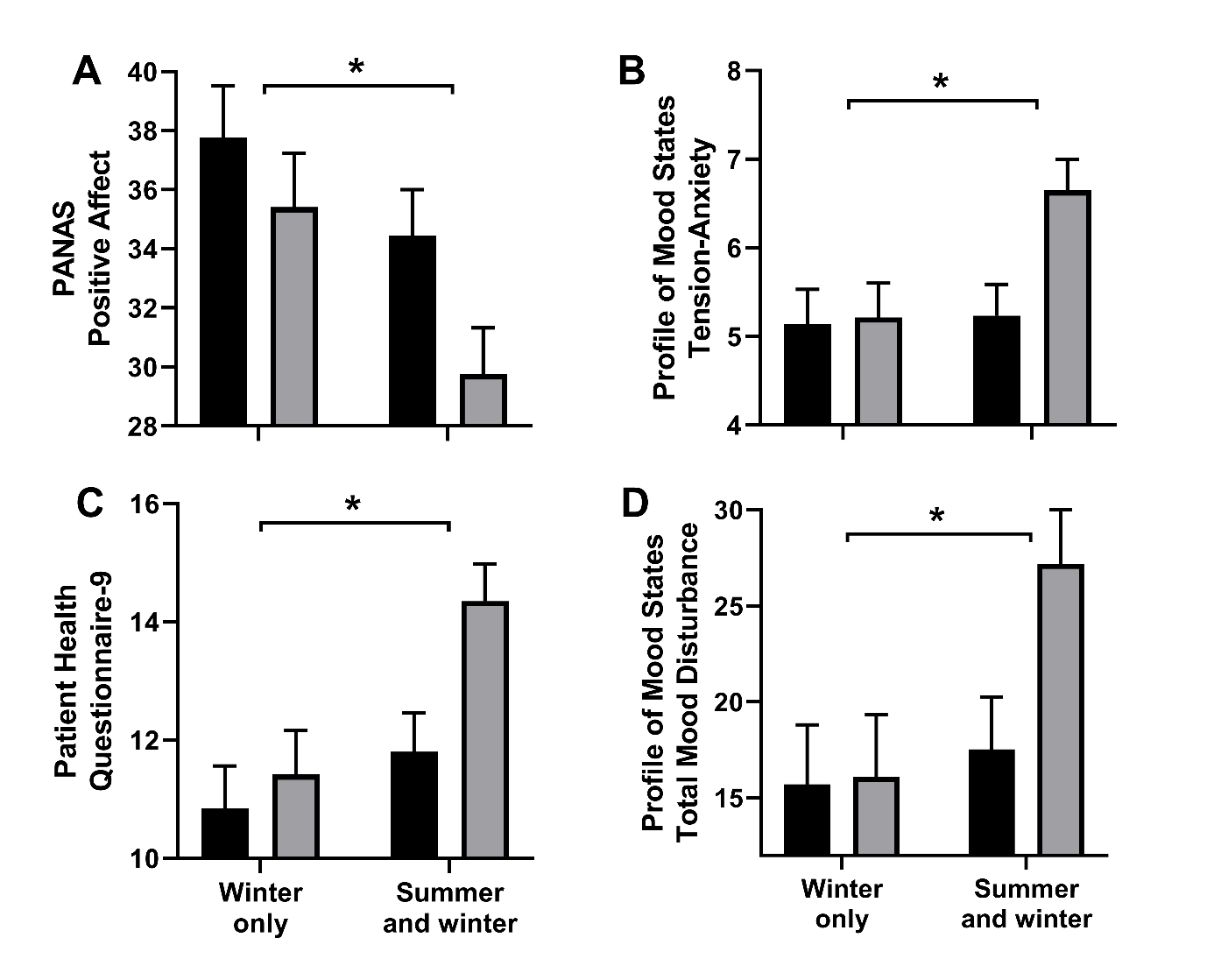
**

**Supplementary Figure 2.** Mean ± SEM scores for (A) positive affect on the Positive and Negative Affect Scale (PANAS), (B) Patient Health Questionnaire depressive symptoms, (C) Profile of Mood States Tension-Anxiety and (D) Profile of Mood States Total Mood Disturbance for expeditioners who arrived on station at the start of the Austral winter (n = 14) compared to expeditioners who had also spent the prior Austral summer in Antarctica (n = 17). Scores are presented for psychological assessments completed early in the winter season (black) and late in the winter season (gray). Astericks represent significant main effects for expedition duration.
